# The Influence of Activated Sludge Augmentation on Its Ability to Degrade Paracetamol

**DOI:** 10.3390/molecules29194520

**Published:** 2024-09-24

**Authors:** Anna Dzionek, Danuta Wojcieszyńska, Ofir Menashe, Daria Szada, Izabela Potocka, Teofil Jesionowski, Urszula Guzik

**Affiliations:** 1 Institute of Biology, Biotechnology and Environmental Protection, Faculty of Natural Science, University of Silesia in Katowice, Jagiellońska 28, 40-032 Katowice, Polandurszula.guzik@us.edu.pl (U.G.); 2 Water Industry Engineering Department, The Engineering Faculty, Kinneret Academic College on the Sea of Galilee, M.P. Emek ’Ha’Yarden, Zemach Junction 15132, Israel; ofirmn@kinneret.ac.il; 3 BioCastle Water Technologies Ltd., Edison Industrial Park, Afikim Jordan Valley 1514800, Israel; 4 Institute of Chemical Technology and Engineering, Poznan University of Technology, Berdychowo 4, 60-965 Poznan, Poland

**Keywords:** paracetamol, *Pseudomonas moorei*, immobilisation, bioaugmentation, activated sludge

## Abstract

Paracetamol is one of the most commonly used painkillers. Its significant production and consumption result in its presence in the environment. For that reason, paracetamol has a negative impact on the organisms living in ecosystems. Therefore, it is necessary to develop effective methods to remove paracetamol from sewage. One of the methods is the bioaugmentation of activated sludge with organisms with increased degradation potential in relation to paracetamol. This study determined the effectiveness of paracetamol degradation by activated sludge augmented with a free or immobilised *Pseudomonas moorei* KB4. To immobilise the strain, innovative capsules made of cellulose acetate were used, the structure of which provides an optimal environment for the development of bacteria. Augmentation with both a free and immobilised strain significantly improves the efficiency of paracetamol biodegradation by activated sludge. Over a period of 30 days, examined systems allowed ten doses of paracetamol decomposition, while the unaugmented system degraded only four. At the same time, using the immobilised strain does not significantly affect the functioning of the activated sludge, which was reflected in the stability of processes such as nitrification. Due to the high stability of the preparation, it can become a valuable tool in wastewater treatment processes.

## 1. Introduction

Paracetamol is one of the most popular drugs used to relieve pain and lower temperature. During its transformation in the body, it may be converted by cytochrome P-450 to the toxic metabolite N-acetyl-p-benzoquinoneimine (NAPQI) [1]. Typically, accumulation of this hepatotoxic metabolite occurs after an uncontrolled high intake. Due to the possibility of transforming paracetamol into a dangerous metabolite, it is crucial to monitor the fate of unmetabolised or partially metabolised paracetamol in the environment. Analysis of paracetamol content in waters on different continents has shown that the highest paracetamol concentrations are found in South America (average 7.6 µg/L). High concentrations are also observed in Africa (5.6 µg/L) and Asia (2.8 µg/L). The lowest concentrations of this drug are observed in Oceania (0.06 µg/L) [2].

One socially acceptable method of removing drugs from the environment is bioaugmentation, using microorganisms with an increased potential to degrade xenobiotics [3]. Among the paracetamol-degrading strains with well-described and well-documented degradative abilities is the *Pseudomonas moorei* KB4 strain. It decomposes paracetamol through 4-aminophenol and then hydroquinone. The latter is cleaved with the participation of an appropriate dioxygenase to aliphatic derivatives, which are incorporated into the central metabolism of the cell [4]. The problem with the use of free cells is their poor survival in the presence of the indigenous microbiome. The solution is to use immobilised bacterial cells. This process allows for an increase in the resistance of such microorganisms to the unfavourable influence of both environmental factors and the impact of other organisms [5].

Promising results were obtained after immobilising the KB4 strain on carriers such as plant sponge or bacterial cellulose, based on the ability of the strain to adsorb on the surface of the carriers [6,7]. After its immobilisation, an increased ability to degrade paracetamol was observed, which was related to the protective effect of the carrier. The formation of a biofilm on its surface limited the toxic impact of 4-aminophenol, an intermediate formed during the degradation of paracetamol [6,7]. Currently, more attention is paid to immobilisation methods that enable microorganisms to be immobilised separately from the external environment. One such method is encapsulation, in which carriers such as gellan gum micro-beads, sodium alginate, carrageenan, polyacrylamide hydrogel, and agarose are used [5,8]. One newly synthesised carrier is the small bioreactor platform (SBP) capsules. The specially designed SBP capsule, 2.5 cm long and 0.8 cm in diameter, separates bacteria from the natural microbiome of the external environment thanks to an external barrier made of a cellulose acetate membrane. The inside of the capsules contains supplemental nutrients in agar and a selected strain. The main task of capsules designed in this way is to increase the degradation potential of the treatment plant environment and to increase the stability of biological processes occurring in sewage during its treatment. This also allows the capsules to protect the introduced strain against dilution during technological processes [8]. This research aimed to use SBP technology in the activated sludge environment to increase paracetamol’s degradation potential. It was important to demonstrate the lack of negative impact of SBP capsules on the functioning of the activated sludge microbiome. This is an essential aspect of research due to the need to maintain the continuity of the technological process of wastewater treatment.

## 2. Results and Discussion

### 2.1. Paracetamol Degradation by Free and Encapsulated KB4 Strains

**Figure 1:**
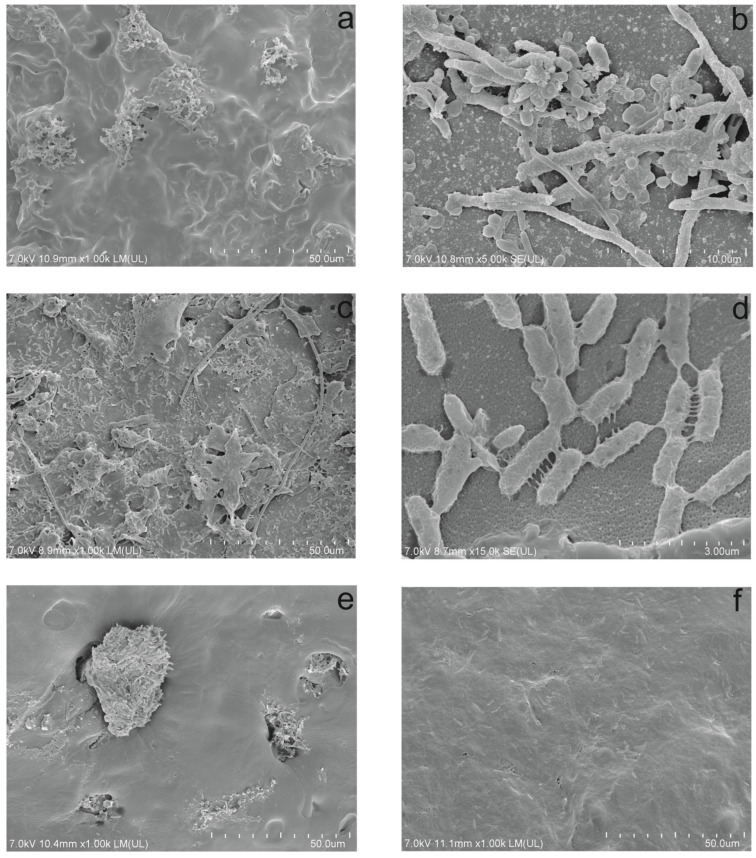
Scanning electron microscopy (SEM) micrographs of the outer (**a**) and inner (**b**) surface of the SBP capsule before degradation tests, the outer (**c**,**d**) and inner (**e**) surface of the SBP capsule after degradation tests, and a cross-section (**f**) of the SBP capsule after degradation tests.

According to literature data, paracetamol is only sometimes completely decomposed in sewage treatment plants, as its degradation involves the formation of highly toxic intermediates. Most often, a very rapid transformation of paracetamol to its first derivative—aminophenol—is observed. However, the latter’s degradation is highly problematic due to its high toxicity to the activated sludge microbiome [3,9,10]. Previous studies indicate that the *Pseudomonas moorei* KB4 strain can fully degrade paracetamol, where the resulting aliphatic hydroxy acids are included in the central metabolism [4]. Studies using the KB4 strain indicate that it decomposes paracetamol, can survive in activated sludge conditions, and retains its degradative abilities after immobilisation [11,12]. So far, the immobilised KB4 strain on a plant sponge or cellulose has been used in degradation studies [6,7]. The ability of the strain to adsorb on the carrier surface was used for this purpose. The disadvantage of these cheap carriers is their biodegradability, which causes their structure to be damaged, and the adsorbed strain can be washed out from the carrier surface in technological systems [13]. In the current studies, an attempt was made to entrap the KB4 strain using SBP capsules made of cellulose acetate. SEM analysis of the carrier indicated that before the studies, the outer side of the SBP capsules was not scolonised with microorganisms (Figure 1a). In contrast, within the inner side of the capsules, scattered cells of the KB4 strain were located between the fibres (Figure 1b). The immobilised strain was introduced into the activated sludge environment. Comparative studies were conducted on 30 mg/L of paracetamol degradation in a bioreactor system. 

**Figure 2:**
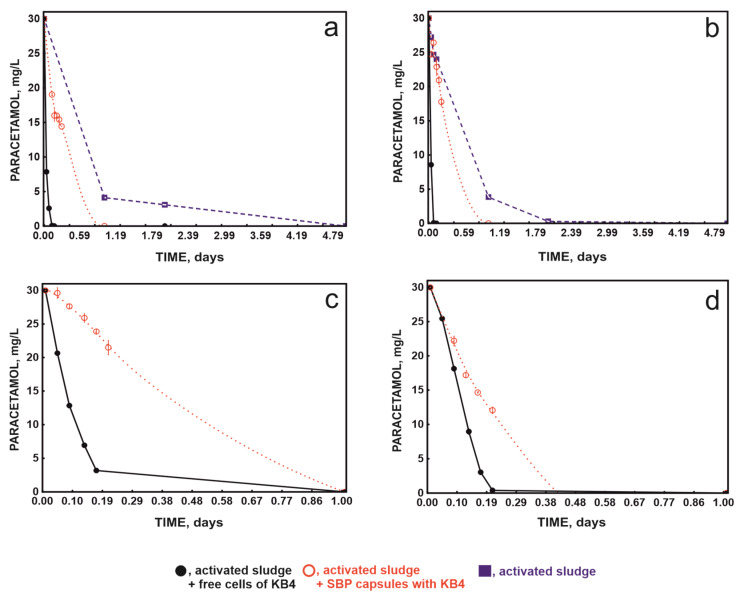
Degradation of dose I (**a**), dose III (**b**), dose VII (**c**), and dose X (**d**) of paracetamol by the studied systems. All experiments were performed in at least three replicates.

To compare the dynamics of the decomposition of 30 mg/L of paracetamol, a 30-day experiment was conducted. During the experiment, successive doses of paracetamol were added to the systems. Paracetamol was introduced in the system with unaugmented activated sludge (control) after the previously introduced dose had utterly degraded. During the experiment, this allowed for the introduction of four doses of paracetamol. In the bioaugmented systems, free or immobilised with the KB4 strain, paracetamol was introduced every 3 days. This allowed for the introduction of 10 doses of paracetamol, each with a concentration of 30 mg/L. It was shown that in each designed system, i.e., the system with activated sludge, the system with activated sludge augmented with the free-strain KB4, and the system with activated sludge augmented with the immobilised-strain KB4, degradation of paracetamol was observed. Figure 2 presents the dynamics of the decomposition of dose I, dose II, dose VII, and dose X of paracetamol introduced into all the tested systems. A detailed analysis of the dynamics of the decomposition of these doses of paracetamol showed that the fastest decomposition of paracetamol occurred in the system enriched with the free strain (Figure 2).

**Figure 3:**
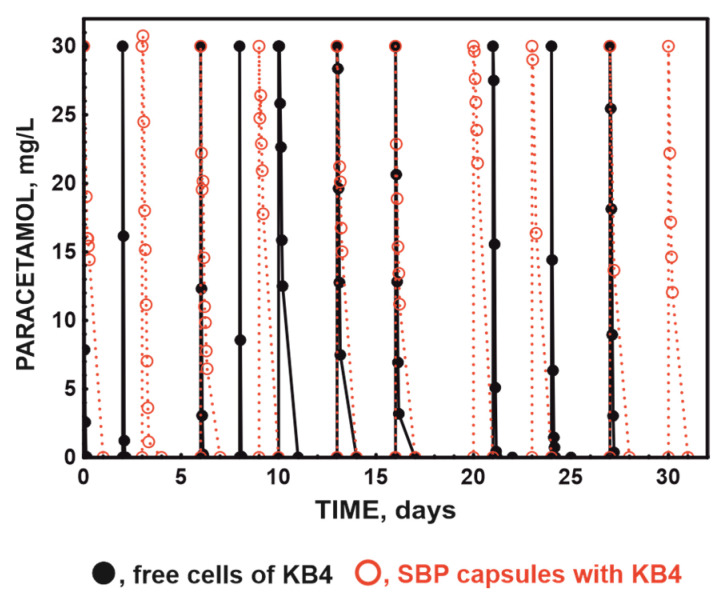
Degradation of ten introduced doses of paracetamol by augmented activated sludge with free- and immobilised-strain KB4.

The slowest decomposition was in the non-augmented system, where during 30 days of this study degradation of only four introduced doses of paracetamol was observed. At the same time, the augmented activated sludge with both the free- and immobilised-strain KB4 decomposed ten doses (Figure 2 and Figure 3). 

The analysis of the dynamics of degradation of subsequent doses in the augmented research systems indicated a comparable rate of all doses of paracetamol degradation by the activated sludge with the free-strain KB4 (Figure 2). In the activated sludge system with SBP capsules, a slow reduction in the degradation time of subsequent doses of paracetamol was observed. This system degraded the seventh dose within 24 h, while the degradation of the tenth dose occurred within 9 h (Figure 2c,d). 

Despite the slower degradation of paracetamol in the bioreactor system with SBP capsules compared to the activated sludge augmented with free KB4 (Figure 3), the encapsulation of the strain can be used in technological processes that require multiple repetitions because the KB4 strain after encapsulation is highly stable. This is reflected in the systematic improvement of degradation properties (Figure 2 and Figure 3). Moreover, in the system bioaugmented with capsules, the biomass used was more than three-hundred-times smaller than in the system bioaugmented with free cells. Hence, in terms of biomass, the observed rate of paracetamol degradation in the system with SBP capsules was higher. For example, the decomposition rate of the first dose of paracetamol in the activated sludge system augmented with free-strain KB4 and SBP capsules was 0.01 mg/h/mg of biomass and 0.63 mg/h/mg of biomass, respectively.

Moreover, efficient degradation using less biomass is of great importance from an economic point of view [14]. In addition, the protective effect of the carrier can protect the strain against the harmful effects of large loads of pollutants burdening sewage treatment plants [5,15]. SBP capsules also enable easier storage of the preparation constructed in this way [8].

SEM analysis of the carrier after paracetamol degradation showed that the outer side of the capsules was scolonised by activated sludge microorganisms (Figure 1c,d). In the obtained micrographs, in addition to bacterial cells, a lipopolysaccharide network was formed (Figure 1d), which probably allows the scolonisation of the outer side of the capsules by activated sludge microorganisms. The changes in the distribution of the *Pseudomonas moorei* KB4 strain on the inner side of the capsule are interesting. SEM micrographs indicate the appearance of functional domains with a high density of the strain (Figure 1e). The presence of KB4 cells is also visible in the cross-sectional photo of the capsule (Figure 1f). It confirms the survival of the KB4 strain during the studies in the bioreactor system. The relatively large cell size of strain KB4 (average 4.23 µm) indicates that the capsule was optimal for growth, and no miniaturisation of the strain was observed, as is usually observed under stressful conditions.

Analysis of intermediates with activated sludge indicated that paracetamol in the unaugmented system was subject to similar changes as the degradation of paracetamol by strain KB4, as the central observed intermediate was hydroquinone [4]. The common degradation pathway indicates the presence of organisms with similar enzymatic systems, which may additionally enhance the directionality of paracetamol degradation [16].

### 2.2. The Impact of Bioaugmentation on the Parameters of Activated Sludge

Maintaining the activated sludge in good condition is extremely important for properly functioning sewage treatment processes. The introduction of large doses of toxins may cause changes in the structure of the sludge and thus significantly affect its functioning. A similar effect may result from introducing additional biological factors into the activated sludge, such as free or immobilised bacterial strains [17]. Therefore, it is crucial to monitor the essential parameters enabling the assessment of the condition of activated sludge, such as the volume index, density index, percentage of water, and mineral and organic content, as well as SOUR, COD, or parameters determining the course of the nitrification process, which is extremely sensitive to changes in the structure of activated sludge organisms and a high concentration of toxins [18].

Analysis of the activated sludge volume index (SVI) collected for testing indicated that initially the sludge had a reduced SVI value (Table 1). 

However, a more than two-fold increase in its value was observed during the tests, indicating that the bioreactor system operated correctly during paracetamol supplementation. The sludge density index (SDI) also showed that the sludge was functioning correctly. Similarly, the SVI value for activated sludge augmented with free cells of the KB4 strain remained acceptable (a decrease of about 30%). The SDI for the tested system was within the limits considered to be the correct level. Also, values such as dry mass content, water content, and mineral and organic contents in the analysed systems indicated the stability of the tested systems (Table 2). 

Introducing capsules to the activated sludge caused a significant increase in the SVI value, which was maintained for 9 consecutive days. The parameter stabilised on day 16 and remained until the end of the experiment. Increased values of this parameter are usually associated with the presence of actinomycetes of the *Nocardia* and *Rhodococcus* genus, which may be caused by a deficiency of one or several essential components, such as oxygen or organic carbon [19,20]. In addition, too-low or too-high concentrations of nitrogen or phosphorus sources may be contributing factors [19,21]. In the studies, glucose was used as a carbon source, which seems to exclude the problem of organic compound deficiency.

Moreover, although the medium was additionally supplemented with ammonium acetate, it should be excluded that the obtained concentrations were the cause of sludge swelling because such an effect was not observed in the two previous systems. The observed destabilisation of the system probably resulted from the use of capsules. The obtained SVI values were correlated with SDI values and total suspended solids content (Table 1). Analysis of SEM images of capsules (Figure 1c) showed that they were a place of surface attachment of actinomycetes, which may indicate that they found a suitable niche for increased multiplication at the initial stage of the experiment. 

However, the long-term conduct of the experiment allowed for stabilising the activated sludge, which consequently translated into the correct parameters of the activated sludge functioning. At the same time, in the system with capsules, no foaming of the activated sludge or formation of scum was observed. At the beginning and end of the experiment, no significant changes in the content of water, mineral, and organic components were also observed (Table 2). The observed slight decrease in dry mass in all systems (Table 2) is probably related, on the one hand, to the negative effect of paracetamol on sensitive activated sludge microorganisms and, on the other hand, to the limited introduced carbon source content. Despite high SVI values, the technological process was not disturbed. The decrease in the SVI parameter is most often associated with the toxic effects of pollutants [22]. Long-term exposure of activated sludge to paracetamol in the presence of capsules did not cause effects associated with the toxic effects of paracetamol, which was reflected in the relatively high SVI. This effect was not observed after augmentation of the activated sludge with the free KB4 strain. This may indicate a positive cooperation between the activated sludge microbiome and the entrapped KB4 strain.

The analysis of the specific oxygen uptake rate (SOUR) indicates its low values for the systems with activated sludge and activated sludge with free-strain KB4. This may be due to the toxicity associated with paracetamol and its degradation intermediates [23]. At the same time, in the system with activated sludge augmented with KB4 capsules, high SOUR values were observed on days 9 and 23, which indicates the high intensity of degradation processes [24]. In turn, the appearance of low SOUR values on days 16 and 31 may result from the temporary accumulation of toxic 4-aminophenol, the degradation of which is slower than paracetamol [4] (Table 3). During the experiments, chemical oxygen demand (COD) decreased significantly from an initially high value, especially in the system with activated sludge with free cells of the KB4 strain. This indicates intensive oxidation processes at the initial stage of the experiment and then a maintained equilibrium of degradation processes (Table 3) [25]. In the activated sludge system with the encapsulated KB4 strain, a relatively high COD level was still observed on the last day of the experiment. This is a surprising result, especially since the initial conditions in all systems were the same, and the high COD level resulted from the presence of paracetamol and its rapidly appearing metabolites. The system with immobilised cells was characterised by the highest metabolic activity during the experiment, as indicated by the SOUR values. No carrier decomposition was observed in the system either, which could explain the increased COD value. Perhaps, despite the integrity of the capsule, the carrier gradually released either temporarily adsorbed paracetamol decomposition intermediates on the carrier or the capsule content diffused, including the remains of the medium and bacterial metabolites. However, more detailed analyses are required to confirm this.

**Figure 4:**
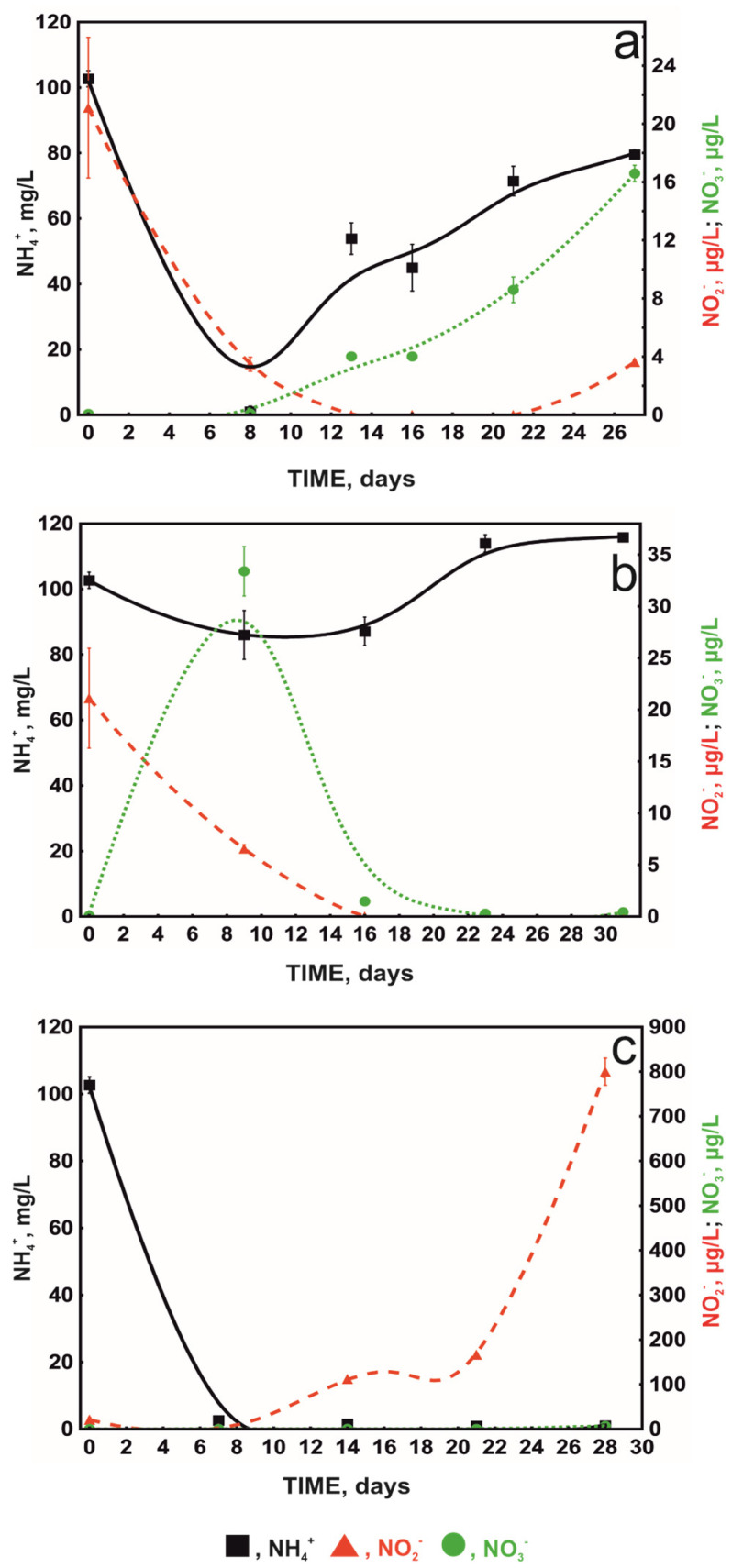
The concentration of NH_4_^+^, NO_3_^−^, and NO_2_^−^ ions during the paracetamol degradation by activated sludge (**a**), activated sludge augmented with free *Ps. moorei* KB4 (**b**), and activated sludge augmented with SBP capsules (**c**). All experiments were performed in at least three replicates.

It is known from the literature that paracetamol in high concentrations above 100 mg/L can inhibit the first stage of nitrification. This is caused by the generation of reactive oxygen species (ROS), which impede ammonium oxidation reactions without affecting the expression of the ammonium monooxygenase gene. On the other hand, lower concentrations below 50 mg/L inhibit the second stage of nitrification, although the nature of this process has not been described [26]. The above observations are consistent with the results obtained for non-augmented activated sludge, where 30 mg/L of paracetamol caused the inhibition of NO_3_^−^ production and the accumulation of nitrite ions (Figure 4a). In contrast to non-augmented activated sludge, the effect was not observed in bioaugmented systems with both free and immobilised KB4 cells (Figure 4b,c). This indicates a positive impact of augmentation on the condition of activated sludge. The faster degradation of paracetamol in these systems probably protects the activated sludge microbiome from the toxic effects of this drug. This is especially true for nitrite-oxidising bacteria (NOB), which are particularly sensitive to the presence of aromatic compounds [27].

## 3. Materials and Methods

### 3.1. Encapsulation of Pseudomonas moorei KB4 in SBP Capsules

Encapsulation of the freeze-dried KB4 bacterium strain was performed by BioCastle Water Technologies Ltd. (Afikim Jordan Valley, Israel) using the SBP technology encapsulation procedure [28]. Briefly, the SBP capsule encases the introduced culture using a cellulose acetate microfiltration membrane (pore size ranging from 0.2 to 0.7 μm), creating a confined 3D environment for the microbial KB4 strain (GenBank KY078305.1) suspension and serving as a physical barrier between the encapsulated bacterial culture and the outer bioreactor medium. The SBP capsules are 2.5 cm long and 0.8 cm in diameter, with a 2 mL internal volume. Each SBP capsule contains approximately 0.1 mg of bacterial powder (dry mass). At this stage, the SBP capsules are in a dry state, and the bacteria are inactive. The dry capsules were submerged for 48–72 h in sterile MSM to activate the encapsulated culture. During that time, the medium penetrated through the membrane and activated the freeze-dried culture while acclimating it to paracetamol consumption as a carbon source.

### 3.2. Designing the Experiment

**Figure 5:**
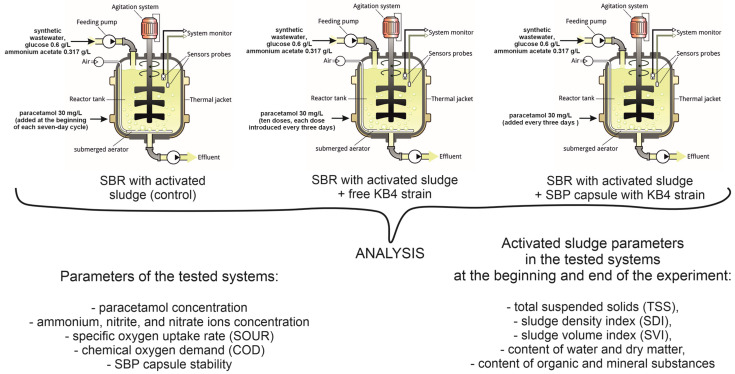
The experimental design.

The experiment was conducted via the Sequencing Batch Reactor (SBR) system in a BIOSTAT A (Sartorius Stedim Biotech, Göttingen, Germany) laboratory bioreactor with a working volume of 5 L, and an automated control of oxygen content (set as 60%), temperature (set as 18 °C), and pH (set as 6.5). The bioreactor was filled with synthetic wastewater (3 L), activated sludge from the wastewater treatment plant Klimzowiec (Chorzów, Poland) (1 L), and KB4 inoculum in the free form (676 mg in dry mass) or encapsulated (20 capsules, 2 mg in dry mass). A control experiment did not include the addition of the KB4 inoculum to the system. The synthetic wastewater was prepared according to Dzionek et al. [13] and was supplemented with paracetamol (30 mg/L) and additional carbon sources like glucose (0.6 g/L) and ammonium acetate (0.317 g/L). One cycle of the SBR lasted for seven days, and after the end of each cycle, activated sludge was sedimented. Half of the synthetic wastewater was replaced by fresh medium, but it was concentrated 2× to maintain the initial concentration of the fresh medium’s minerals, carbon, and nitrogen sources. Paracetamol was added every three days (main experiments) or at the beginning of each cycle (control experiment), and glucose was supplemented every three days. The experiment lasted 30 days, during which 10 doses of paracetamol were introduced. The experimental design is shown in Figure 5.

### 3.3. Biochemical Analysis

The metabolic activity of activated sludge was determined every seventh day of the cycle by the specific oxygen uptake rate (SOUR) via an Elmetron multiparameter equipped with a Clark electrode, according to the US Environmental Protection Agency [29], with some minor modifications. A total of 5 ml of the activated sludge was introduced into a 50 mL biochemical oxygen demand bottle (BOD) containing 25 mL of fresh aerated synthetic wastewater, and a Clark electrode was immediately inserted into the bottle. After the content was isolated from the atmosphere, the dissolved oxygen (DO) readings were performed every 60 s for 10 min or until the DO dropped below 1 mg/L. Preparation of the plot DO (mg/L) versus time (min) allowed the determination of the slope of the linear portion of the curve, which was used to calculate the SOUR according to the following equation:

(1) $$SOUR=\frac{\frac{{DO}_{0}-{DO}_{n}}{t_{n}-t_{0}}}{TSS}$$

SOUR—specific oxygen uptake rate (mg/L∗min)
DO_0_—dissolved oxygen at the beginning of the linear portion of the curve (mg/L)
DO_n_—dissolved oxygen at the end of the linear portion of the curve (mg/L)
t_n_—time at the end of the linear portion of the curve (min)
t_0_—time at the beginning of the linear portion of the curve (min)
TSS—total suspended solids in the sample (g/L)

The total suspended solids in the sample were calculated according to the standard filtration method [30]. 

Every seventh day of the cycle, parameters were also determined such as chemical oxygen demand (COD), and the content of the ammonium ion, nitrites (III), and nitrates (V). For assays, collected samples were firstly centrifuged (14,000 rpm, 15 min, 4 °C) to discard particulate matter and biomass. COD was analysed using the potassium dichromate method using standard procedures [30]. Determination of the ammonium ion content was carried out using the Nessler method [31]. The nitrites (III) content was determined using the colourimetric naphthylamine method [32]. The content of nitrates (V) was evaluated by the Brucine method [33].

#### 3.3.1. HPLC Analysis of Paracetamol

Paracetamol decomposition was monitored by RP-HPLC (Merck HITACHI, Darmstadt, Germany) equipped with an Ascentis Express^®^ C18 HPLC Column (100 × 4.6 mm), pre-column Opti-Solw^®^ EXP, and a DAD detector (Merck HITACHI, Darmstadt, Germany). The mobile phase for paracetamol separation consisted of acetonitrile and acetic acid 1% (5:95 *v*/*v*) with a 1 mL/min flow rate. Paracetamol was detected at wavelength 240 nm. Identification and quantification were conducted by comparing the HPLC retention times and UV–visible spectra with the external standards.

#### 3.3.2. Analysis of Activated Sludge Parameters

In order to assess the condition of the activated sludge at the beginning of each experiment and the end of each cycle, parameters like total suspended solids (TSS), sludge density index (SDI), sludge volume index (SVI), and the content of water, dry matter, and organic and mineral substances were evaluated. The parameters mentioned were calculated according to the standard methods [30].

#### 3.3.3. GC-MS Analysis of Intermediates

Culture samples (40 mL) were collected and centrifuged. For qualitative analysis, Agilent 1290 Infinity UHPLC coupled to a 6546 Quadrupole Time of Flight Mass Spectrometer with a Dual AJS ESI source operated at positive ion mode (Agilent Technologies, Santa Clara, CA, USA) was used.

The chromatographic separation of analytes was accomplished using a Poroshell EC-C18 column, 2.1 × 50, 1.9 µm (Agilent Technologies, Santa Clara, CA, USA), maintained at 35 °C during the analysis. The mobile phase consisted of 10 mM ammonium formate in water containing 0.1% formic acid (A) and methanol (B). The gradient to elution used was: 0 min: 5% B, 0.5–25 min: 5–95% B, 25–29 min: 95% B, and 30 min: 5% B. The volume of sample injected was 2 µL. The column conditioning time after analysis was 4 min. 

The Dual AJS ESI source parameters were as follows: drying gas temperature, 300 °C; drying gas flow, 9 L/min; nebuliser pressure, 40 psi; sheath gas temp, 350 °C; sheath gas flow, 11 L/min; capillary entrance voltage, 4000 V; and nozzle voltage, 500 V. On the base, the accuracy of the mass measurement was <5 ppm and the isotopic distribution of the compounds was >95%; metabolites were also identified.

### 3.4. SEM Analysis

The structure of the capsule’s surface and internal matrix was observed at the start and the end of the experiment using scanning electron microscopy according to Dzionek et al. [34], with some modifications. Samples were firstly fixed in glutaraldehyde 3% for 24 h and dehydrated with ethanol (30, 50, 70, 80, 90, 95, and 100%, each for 10 min). Next, samples were frozen in liquid nitrogen, lyophilised, and sputter-coated in a Safematic CCU-010 HV SEM Coating System (Safematic GmbH, Zizers, Switzerland) with a thin film of gold and observed with a Hitachi SU8010 field-emission scanning electron microscope (Hitachi High-Technologies Corporation, Tokyo, Japan).

## 4. Conclusions

In summary, this study has shown that augmentation is a suitable method for increasing the efficiency of paracetamol decomposition in activated sludge systems. Such augmentation can be performed with both a free and immobilised strain. The decrease in COD values in the activated sludge system with free cells of the KB4 strain indicates intensive oxidation processes in the initial phase of the experiment, leading to the equilibrium state of degradation processes.

The rate of paracetamol degradation is directly related to the amount of paracetamol-degrading biomass within the treatment system. Consequently, our findings indicate that the unaugmented system has the lowest levels of paracetamol-degrading biomass (excluding KB4), whereas, among the augmented systems, the free KB4 system contains the highest amount of paracetamol-degrading biomass. Considering the total loss of *Pseudomonas moorei* KB4 strain biomass during the exchange of activated sludge and the costs associated with the need to multiply it again, the solution using the immobilised strain is better from an economic point of view. The SBP capsules themselves provide optimal living conditions for the immobilised strain and, at the same time, can be recovered after the technological process is completed.

## Figures and Tables

**Table 1:** Parameters of activated sludge during the experiment.

Experimental System	Time (Days)	Sludge Volume Index (mL/g)	Sludge Density Index (100/SVI)	Total Suspended Solids (mg/L)
Activated sludge	Sludge output parameters	40.47	2.47	5140.00
	0	40.47	2.47	1285.00
	7	83.33	1.20	1200.00
	14	101.69	0.98	1180.00
	21	57.55	1.74	2780.00
	28	85.31	1.17	2110.00
Activated sludge with free KB4 strain	Sludge output parameters	86.79	1.15	10,600.00
	0	86.79	1.15	2650.00
	8	86.33	1.16	1390.00
	16	69.44	1.44	2880.00
	21	60.79	1.65	3290.00
	27	60.61	1.65	3300.00
Activated sludge with SBP capsules with KB4 strain	Sludge output parameters	167.95	0.60	3453.33
	0	167.95	0.60	863.33
	9	169.49	0.59	590.00
	16	99.01	1.01	1010.00
	23	125.00	0.80	1280.00
	31	129.03	0.78	1240.00

**Table 2:** Parameters of dry matter, water, and mineral and organic content at the beginning and end of the experiment.

Experimental System	Parameters	Start of the Experiment	End of the Experiment
Activated sludge	Dry matter content (%)	0.54 ± 0.00	0.34 ± 0.01
	Water content (%)	99.46 ± 0.00	99.66 ± 0.01
	Mineral content (%)	29.16 ± 1.83	34.03 ± 0.80
	Organic content (%)	70.84 ± 1.83	65.97 ± 0.80
Activated sludge with free KB4 strain	Dry matter content (%)	0.89 ± 0.08	0.37 ± 0.01
	Water content (%)	99.11 ± 0.08	99.63 ± 0.01
	Mineral content (%)	26.14 ± 0.67	25.93 ± 0.83
	Organic content (%)	73.86 ± 0.67	74.07 ± 0.83
Activated sludge with SBP capsules with KB4 strain	Dry matter content (%)	0.31 ± 0.04	0.29 ± 0.04
	Water content (%)	99.69 ± 0.04	99.71 ± 0.04
	Mineral content (%)	31.11 ± 2.34	32.01 ± 2.70
	Organic content (%)	68.89 ± 2.34	67.99 ± 2.70

**Table 3:** Specific oxygen uptake rate (SOUR) and chemical oxygen demand (COD) during the experiment.

Experimental System	Time (Days)	SOUR (mg/g∗h)	COD (mg O_2_/L)
Activated sludge	0	2.59 ± 0.60	1232.00 ± 31.00
	7	5.39 ± 0.86	176.00 ± 31.00
	14	1.50 ± 0.70	99.00 ± 47.00
	21	1.20 ± 0.24	110.00 ± 31.00
	28	1.10 ± 0.37	0.00 ± 0.00
Activated sludge with free KB4 strain	0	2.45 ± 0.00	1232.00 ± 31.00
	8	1.24 ± 0.38	242.00 ± 16.00
	16	1.10 ± 0.03	165.00 ± 31.00
	21	1.20 ± 0.19	0.00 ± 0.00
	27	1.64 ± 0.00	0.00 ± 0.00
Activated sludge with SBP capsules with KB4 strain	0	3.30 ± 0.53	1232.00 ± 31.00
	9	11.15 ± 2.50	176.00 ± 31.00
	16	4.55 ± 0.08	220.00 ± 62.00
	23	13.87 ± 2.27	286.00 ± 0.00
	31	3.62 ± 0.54	253.00 ± 47.00

## Data Availability

The data presented in this study are available on request from the corresponding author.

## References

[1] Dargue R., Zia R., Lau C., Nicholls A.W., Dare T.O., Lee K., Jalan R., Coen M., Wilson I.D. (2020). Metabolism and effects on endogenous metabolism of paracetamol (acetaminophen) in a porcine model of liver failure. Toxicol. Sci..

[2] Wilkinson J.L., Boxall A.B.A., Kolpin D.W., Leung K.M.Y., Lai R.W.S., Galban-Malagon C., Adell A.D., Mondon J., Metian M., Marchant R.A. (2022). Pharmaceutical pollution of the world’s rivers. Proc. Natl. Acad. Sci. USA.

[3] Palma T., Valentine J., Gomes V., Faleiro M., Costa M. (2022). Batch studies on the biodegradation potential of paracetamol, fluoxetine and 17α-ethinylestradiol by the *Micrococcus yunnanensis* strain TJPT4 recovered from marine organisms. Water.

[4] Żur J., Wojcieszyńska D., Hupert-Kocurek K., Marchlewicz A., Guzik U. (2018). Paracetamol—Toxicity and microbial utilisation. *Pseudomonas moorei* KB4 as a case study for exploring degradation pathway. Chemosphere.

[5] Najim A.A., Radeef A.Y., al-Doori I., Jabbar Z.H. (2024). Immobilization: The promising technique to protect and increase the efficiency of microorganisms to remove contaminants. J. Chem. Technol. Biotechnol..

[6] Surma R., Wojcieszyńska D., Karcz J., Guzik U. (2021). Effect of *Pseudomonas moorei* KB4 cells’ immobilisation on their degradation potential and tolerance towards paracetamol. Molecules.

[7] Żur J., Piński A., Michalska J., Hupert-Kocurek K., Nowa K.A., Wojcieszyńska D., Guzik U. (2020). A whole-cell simmobilisation system on bacterial cellulose for the paracetamol-degrading *Pseudomonas moorei* KB4 strain. Int. Biodeter. Biodegrad..

[8] Menashe E., Kurzbaum E. (2014). Small-bioreactor platform technology as a municipal wastewater additive treatment. Water Sci. Technol..

[9] Chopra S., Kumar D. (2023). Characterization and biodegradation of paracetamol by biomass of *Bacillus licheniformis* strain PPY-2 isolated from wastewater. Rend. Lincei. Sci. Fis. Nat..

[10] Chacón F.J., Cayuela M.L., Sanchez-Monedero M.A. (2022). Paracetamol degradation pathways in soil after biochar addition. Environ. Pollut..

[11] Marchlewicz A., Guzik U., Hupert-Kocurek K., Wojcieszyńska D. (2023). Evaluation of the defined bacterial consortium efficacy in the biodegradation of NSAIDs. Molecules.

[12] Nowak A., Dzionek A., Wojcieszyńska D., Guzik U. (2023). Application of simmobilised biocatalysts in the biotransformation of non-steroidal anti-inflammatory drugs. Appl. Sci..

[13] Dzionek A., Wojcieszyńska D., Adamczyk-Habrajska M., Guzik U. (2020). Enhanced degradation of naproxen by simmobilisation of *Bacillus thuringiensis* B1 (2015b) on loofah sponge. Molecules.

[14] Martinez-Hernandez E., Amezcua-Allieri M.A., Aburto J. (2021). Assessing the cost of biomass and bioenergy production in agroindustrial processes. Energies.

[15] Ferreira R.M., Ribeiro B.D., Stapelfeldt D.M.A., do Nascimento R.P., Moreira M.F.R. (2023). Oil biodegradation studies with an simmobilised bacteria consortium in plant biomass for the construction of bench-scale bioreactor. Clean. Chem. Eng..

[16] Tahri N., Bahafid W., Sayel H., El Ghachtouli N. (2013). Biodegradation: Involved Microorganisms and Genetically Engineered Microorganisms.

[17] Michalska J., Piński A., Żur J., Mrozik A. (2020). Analysis of the bioaugmentation potential of *Pseudomonas putida* OR45a and *Pseudomonas putida* KB3 in the sequencing batch reactors fed with the phenolic landfill leachate. Water.

[18] Woznica A., Nowak A., Karczewski J., Klis C., Bernas T. (2010). Automatic biodetector of water toxicity (ABTOW) as a tool for examination of phenol and cyanide contaminated water. Chemosphere.

[19] Janczukowicz W., Szewczyk M., Krzemieniewski M., Pesta J. (2001). Settling properties of activated sludge from a sequencing batch reactor (SBR). Pol. J. Environ. Stud..

[20] Tsang Y.F., Sin S.N., Chua H. (2008). *Nocardia* foaming control in activated sludge process treating domestic wastewater. Biores. Technol..

[21] Wongburi P., Park J.K. (2022). Prediction of sludge volume index in a wastewater treatment plant using recurrent neural network. Sustainability.

[22] Cui K., Xu Q., Sheng X., Meng Q., Shang G., Ma Y., Zhang Z., Guo K. (2021). The impact of bioaugmentation on the performance and microbial community dynamics of an industrial-scale activated sludge sequencing batch reactor under various loading shocks of heavy oil refinery wastewater. Water.

[23] Du Y., Chen Y., Zou L., Deng S., Li G., Zhang D. (2019). Monitoring the activated sludge activities affected by industrial toxins via an early-warning system based on the relative oxygen uptake rate (ROUR) Index. Appl. Sci..

[24] Kim I.S., Young J.C., Kim S., Kim S. (2001). Development of monitoring methodology to fingerprint the activated sludge processes using oxygen uptake rate. Environ. Eng. Res..

[25] Dai W., Pang J.-W., Ding J., Wang Y.-Q., Zhang L.-Y., Ren N.-Q., Yang S.-S. (2023). Study on the removal characteristics and degradation pathways of highly toxic and refractory organic pollutants in real pharmaceutical factory wastewater treated by a pilot-scale integrated process. Front. Microbiol..

[26] Park S., Oh S. (2020). Inhibitory mechanisms and fate of the analgesic drug acetaminophen in nitrifying activated sludge. J. Hazard. Mater..

[27] Peng Y., Zhu G. (2006). Biological nitrogen removal with nitrification and denitrification via nitrite pathway. Appl. Microbiol. Biotechnol..

[28] Menashe O. Microorganism Comprising Particles and Uses of Same. Patent application number.

[29] U.S. Environmental Protection Agency, Office of Water, Office of Science and Technology Engineering and Analysis Division (2001). METHOD 1683: Specific Oxygen Uptake Rate in Biosolids.

[30] APHA (1998). Standard Methods for the Examination of Water and Wastewater.

[31] Jeong H., Park J., Kim H. (2013). Determination of NH_4_^+^ in environmental water with interfering substances using the modified Nessler method. J. Chem..

[32] Sen N.P., Donaldson B. (1978). Improved colorimetric method for determining nitrate and nitrite in foods. J. Assoc. Off. Anal. Chem..

[33] Petriconi G.L., Papee H.M. (1971). On routine colorimetric determination of trace nitrates, by brucine, in the presence of chloride. Water Air Soil Pollut..

[34] Dzionek A., Wojcieszyńska D., Hupert-Kocurek K., Adamczyk-Habrajska M., Guzik U. (2018). Immobilization of *Planococcus* sp. S5 strain on the loofah sponge and its application in naproxen removal. Catalysts.

